# Preference and willingness to receive non-communicable disease services from primary healthcare facilities in Bangladesh: A qualitative study

**DOI:** 10.1186/s12913-022-08886-3

**Published:** 2022-12-03

**Authors:** Ashraful Kabir, Nazmul Karim, Baki Billah

**Affiliations:** Department of Epidemiology and Preventive Medicine, School of Public Health and Preventive Medicine, Monash University, Melbourne, Australia

**Keywords:** Non-communicable diseases, Preference and willingness, Primary healthcare facilities, Social-ecological model

## Abstract

**Background:**

In Bangladesh, non-communicable diseases (NCDs) are increasing rapidly and account for approximately 68% of mortality and 64% of disease burden. NCD services have been significantly mobilized to primary healthcare (PHC) facilities to better manage the rising burden of NCDs. However, little is known about community members’ preference and willingness to receive NCD services from PHC facilities; therefore, this particular subject is the focus of this study.

**Methods:**

A qualitative study was conducted from May 2021 to October 2021. Data were collected via 16 focus group discussions involving community members and 14 key informant interviews with healthcare professionals, facility managers, and public health practitioners. Based on a social-ecological model (SEM), data were analyzed thematically. The triangulation of methods and participants was conducted to validate the information provided.

**Results:**

Preference and willingness to receive NCD services from PHC facilities were influenced by a range of individual, interpersonal, societal, and organizational factors that were interconnected and influenced each other. Knowledge and the perceived need for NCD care, misperception, self-management, interpersonal, and family-level factors played important roles in using PHC facilities. Community and societal factors (i.e., the availability of alternative and complementary services, traditional practices, social norms) and organizational and health system factors (i.e., a shortage of medicines, diagnostic capacity, untrained human resources, and poor quality of care) also emerged as key aspects that influenced preference and willingness to receive NCD services from PHC facilities.

**Conclusion:**

Despite their substantial potential, PHC facilities may not take full advantage of managing NCDs. All four factors need to be considered when developing NCD service interventions in the primary healthcare system to better address the rising burden of NCDs.

## Background

Globally, the gradual increase of non-communicable diseases (NCDs) has become a major public health concern. In 2021, NCDs were accountable for 41 million deaths (71% of all deaths), and 77% of deaths occurred in low- and middle-income countries (LMICs) [1, 2]. Like many LMICs, Bangladesh is experiencing a rapid increase in NCDs due to socio-demographic and epidemiological transmissions [3]. In 2016, estimated deaths from NCDs were 856,000 (67% of total deaths) in Bangladesh, which shared approximately 64% of the disease burden [4]. Furthermore, it is assumed that the number of deaths and disabilities from NCDs will likely increase in the coming years due to individual, contextual, and health system factors [3, 5, 6], including the growing number of elderly populations, tobacco use, consumption of alcohol, physical inactivity, and unhealthy diets. Rapid urbanization will also probably result in the gradual increase of NCDs in different age groups (e.g., relatively young people), occupational groups, and geographical locations (e.g., rural and urban slums) [7, 8].

Recognizing the current NCD burden and future predictions, a dozen of global declarations and strategies have been proposed to prevent and control NCDs [9–12]. In line with these global commitments and strategies, many countries and territories have designed a wide range of interventions to address the NCD burden. As a common strategy, NCD services and treatments were significantly mobilized to strengthen health systems, and many systems used the World Health Organization’s package of essential noncommunicable (WHO–PEN) disease interventions to provide comprehensive, need-oriented, and quality NCD services to the population at the primary healthcare level. The underlying purpose of mobilizing supplies and resources is to increase the coverage and utilization of affordable and quality NCD services as these are fundamental to effective intervention. Highlighting the importance of community preference and willingness to access targeted healthcare interventions, studies conducted in China [13–16], South Africa [17], and El Salvador [18] reported a range of attributes and factors in individual, community, organization, and policy levels that influenced the preference and willingness to access NCD services and care.

Aligning to the aforementioned global goals and national priorities, the Bangladesh Government is committed to address the rising burden of NCDs. As a controlling strategy, the country emphasized strengthening the primary healthcare system, which is the first point of contact for around 70% of the population. An extended package of recommended NCD services and care has been made available in PHC facilities to increase the access and utilization of services in the community [19]. The availability and supply of resources, such as the healthcare workforce, technology, equipment, medicine and amenities, and infrastructure facilities have been increased, which is a critical first step to better managing the NCD burden [5, 19]. However, there is a dearth of information about how and to what extent communities prefer and are willing to receive NCD services from PHC facilities. Although a few studies have examined the health-seeking behavior of an adult population with NCDs in urban slums [20] and the feasibility of the integrated management of diabetes and hypertension in PHC facilities [21], a complete and comprehensive investigation of factors influencing the preference and willingness to receive NCD services from PHC facilities remain a priority.

Using a social-ecological model (SEM), this study is designed to provide a complete and holistic picture of community members’ preferences and willingness to receive the NCD services available in PHC facilities. The findings may be useful in understanding community perspectives of NCD interventions, which are essential components of a well-functioning healthcare system in Bangladesh and similar settings elsewhere.

## Methods and materials

### Study design

An exploratory qualitative study design was used.

### Study time and settings

This study was conducted between May and October 2021 in Bangladesh. To understand and explore the factors influencing the perception, practice, preference, and willingness of community members to receive NCD services from PHC facilities, data were collected from four administrative districts in Bangladesh: Cumilla, Jhenaidah, Rajshahi, and Sylhet. These selected areas are situated in the rural settings of the country. Bangladesh's health system is noticeably uniform concerning the health service delivery, organization of the healthcare professionals, and infrastructure and logistics supply. However, varying socio-demographic features, geographical elements, livelihood patterns, sociocultural norms, and practices may affect health outcomes. Taking these variabilities into account data were purposefully collected from participants with a wide variety of roles, backgrounds, organizations, and geographical locations. It is worth that Bangladesh's health system is pluralistic—multiple providers are applying a mixed system of medical practices under the Ministry of Health and Family Welfare from primary to tertiary levels [22]. Bangladesh’ Corresponding o the administrative structure, Bangladesh has approximately 87,310 villages, 40,977 wards, 4553 Union, 490 sub-districts, 64 districts, 4 metropolitan cities, and 8 divisions [23]. Primary healthcare services are established at the sub-district (locally known as Upazila) level, mostly provided through various Upazila-based healthcare facilities and frontline staff. Approximately 70% of the total population of the country receives healthcare from the primary healthcare system as the first-line contact for health needs [22, 24]. In parallel, the primary healthcare services in the metropolitan and municipalities areas are mainly organized through project-based targeted interventions and partnership programs between local governments (i.e., municipalities, and city corporations), NGOs, and donor agencies under the Ministry of Local Government, Rural Development & Cooperation [25, 26]. Due to various structural and operational factors such as complex healthcare delivery system, target population (i.e., highly focused on the slums dwellers, poor neighborhoods), greater reliance on the private healthcare sector, resource mobilization and allocation modality, relatively small coverage, and study budget and field constraints, we decided to exclude metropolitan cities in this study as detailed in our protocol paper [22].

### Study participants and sampling strategy

Sixteen focused group discussions (FGDs) were conducted involving people in the community who had developed at least one of the following major NCD: cervical cancer, chronic respiratory disease (CRI), cardiovascular diseases (CVDs), and diabetes mellitus (DM). Fourteen key informant interviews (KIIs) were also conducted with healthcare providers, facility managers, and public health practitioners. The participants were purposefully selected, which is largely practiced in qualitative research and has previously been used in the context of Bangladesh [27, 28]. In this process, we first approached potential participants and explained the study’s aims, objectives, and expectations. The participants were selected based on criteria such as (i) people aged 18 years and above, (ii) time and availability, and (iii) volunteer participation. The number of interviews (sample size) was determined following the principle of data saturation, i.e., the interviews were completed when no further new information, dimensions, or concepts emerged, according to the proposition by Guest et al. [29]. A stepwise procedure was followed to reach the point of data saturation. This involved multiple interviewers concurrently conducting interviews and then performing initial/axial coding. After two-thirds of the interviews were conducted, the research team independently developed the codes and checked that no new information, dimensions, or themes emerged. When the research team agreed that no new themes were being generated, data saturation was achieved. However, it is worth mentioning that a few basic norms were followed in selecting the participants in terms of: (i) ensuring there was a maximum variation of organizations, age, sex, occupations, income groups, (ii) an iterative process (moving back and forth between data collection and coding), and (iii) reflexivity (assessed self-roles of the interviewers). Semi-structured question guidelines were used in the interviews, which were piloted and tested in a similar setting (outside the original field sites).

### Data collection procedure

The interviews were conducted in Bangla, the native language of both the interviewers and participants. The interviews were conducted at their homes or workplace and were audio-recorded with the permission of the interviewees. Two people (social science and medicine graduates) conducted the interviews, and two research assistants (social science graduates) captured the field notes. The interviewers were trained in qualitative research methods and experienced in applying various data collection tools and techniques in similar settings. Each FGD took approximately 45–60 minutes, and KIIs took an average of 30–45 minutes. Before starting discussions, the research team built a good rapport with the participants and described the purpose of the study. Each interviewee was informed about the recording, and written consent was obtained before starting the interviews. This process lasted for around one hour with each participant.

### Data analysis and theoretical framework

**Fig. 1:**
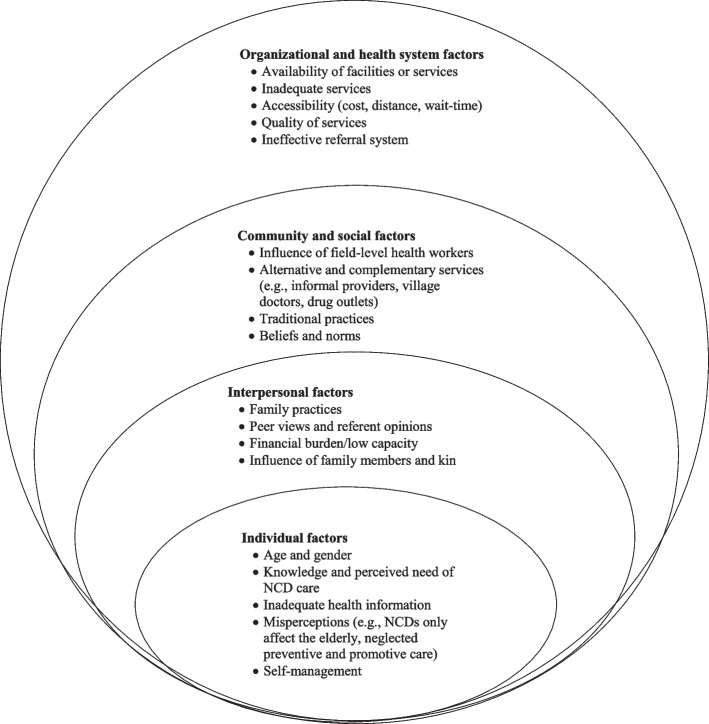
Social-ecological framework on the preference and willingness to use NCD services at PHC facilities

Based on the SEM, data were thematically analyzed (Figure 1). SEM is a theory-based conceptual framework that considers a wide range of factors at individual, personal, societal, and organizational levels, which are interconnected and interact with each other within the wider social system [30]. Thus, the SEM adequately facilitates the dynamic interplay of individual, interpersonal, social, and organizational factors that produce behavioral outcomes regarding the preference and willingness to receive NCD-related services and care from PHC facilities. Guided by the SEM and research objectives, a hybrid approach combining deductive and inductive coding procedures was applied to identify the themes related to the perception, practice, and willingness to receive NCD services and care in the primary healthcare system. The ‘consolidated criteria for reporting qualitative studies (COREQ): 32-item checklist’ was followed to report the findings [31]. Initially, a list of probable codes was developed based on the information available in the literature and the understanding and experiences of the research team members that have been developed through their engagement in their respective study domains. Initially, three researchers repeatedly read the transcription line-by-line and coded it in line with the predefined set of codes [32]. Concurrently, additional codes were identified to fit the relevant information/statements obtained during the reading of the transcriptions. The final codebook was developed by combining them. In the next step, a few clusters were searched based on the similarities of the identified codes. In the final stage, several themes emerged to report the findings. The independent coders maintained formal discussions to resolve any disagreements so that a consensus was reached.

## Results

### Characteristics of the participants

This section presents the socio-demographic profiles of the participants and is followed by the thematic analysis. Table 1 shows that the combined mean age for FGD participants (*n* = 92) was 51 years (standard deviation (SD) ±8). Nearly one-third (33/92) of the participants did not have formal schooling, and only seven participants had above grade 10 (X) schooling. Most of the participants across the sites were married (61/92), and the majority of the participants lived in a joint family setting (69/92). Informal occupations (e.g., agricultural labor, homemakers, students, businessmen, farmers, etc.) were predominant across sites (79/92). Nearly two-thirds (74/92) were Muslim. The highest number of participants (52/92) had a monthly family income of >10,000 BDT (87 BDT ≅ 1 USD). More than half (52/92) of the participants had multiple NCDs (e.g., chronic respiratory illness, cancer, cardiovascular disease, and diabetes), and the majority of the participants had suffered from NCDs for over five years.

**Table 1:** Socio-demographic characteristics of participants (participants: *n=*92; FGD: *n=*16)

Variables	Study sites				Combined
	Cumilla	Jhenaidah	Rajshahi	Sylhet	
**Age in years (mean ± SD)**	55 (± 12)	54 (± 10)	49 (± 5)	43 (± 11)	51 (± 8)
**Gender** ***(n)***					
Male *(n)*	9	10	12	10	41
Female *(n)*	11	15	14	11	51
**Education** ***(n)***					
No formal education *(n)*	10	9	7	7	33
Primary school (I–V grade) *(n)*	4	8	9	11	32
Secondary school (VI–X grade) *(n)*	4	8	5	3	20
Higher (> X grade) *(n)*	2	-	5	-	7
**Marital status** ***(n)***					
Married *(n)*	16	16	14	15	61
Unmarried *(n)*	3	4	5	2	14
Widowed *(n)*	1	5	7	4	17
**Family type (*n)***					
Nuclear *(n)*	5	10	2	6	23
Joint *(n)*	15	15	24	15	69
**Occupations** ***(n)***					
Formal *(n)*	4	3	2	4	13
Informal *(n)*	16	22	24	17	79
**Religious identity** ***(n)***					
Muslim *(n)*	16	25	17	16	74
Hinduism *(n)*	4	-	6	4	14
Others *(n)*	-	-	3	-	3
**Income** ***(n)***					
< 5000 tk *(n)*	-	4	5	-	9
5,000–10,000 tk *(n)*	4	11	15	5	35
> 10,000 tk *(n)*	16	10	6	16	48
**NCD status** ***(n)***					
Developed at least 1 NCD *(n)*	6	14	11	9	40
Multiple NCDs *(n)*	14	11	15	12	52
**Length of suffering from NCDs** ***(n)***					
< 3 years *(n)*	3	3	2	2	10
3–5 years *(n)*	9	3	3	7	22
> 5 years *(n)*	8	19	21	12	60

Table 2 presents some basic background information about the fourteen KII participants. The mean age of the KII participants (*n* = 14) was 42 years (SD ±9), and of them nine were male. The majority of KII participants were healthcare providers (8/14), and the remainder were involved in policy planning (2/14) or healthcare activism (2/14).

**Table 2:** Background characteristics of KII participants (*n=*14)

Characteristics	Values
**Age in years (mean ± SD)**	42 (±9)
**Gender** ***(n)***	
Male *(n)*	9
Female *(n)*	5
**Type of provider** ***(n)***	
Healthcare professional *(n)*	8
Healthcare manager *(n)*	2
Policy planner/independent consultant *(n)*	2
Civil society/NGO workers *(n)*	2
**Length of service/experience** ***(n)***	
< 5 years *(n)*	3
6–10 years *(n)*	4
> 10 years *(n)*	7

### Thematic analysis

Dominant themes were identified and grouped into categories reflecting the major interrelated elements of the SEM (Figure 1). We found four major themes: (i) individual factors; (ii) interpersonal and family factors; (iii) community and social factors; (iv) organizational and health system factors. Each of the major themes was further divided into several sub-themes.

### Individual factors

Participants from all sites involved in the FGDs and KIIs revealed that preference and willingness to receive NCD services from PHC facilities were influenced by a wide array of individual factors, including age and gender, knowledge, health information, misperception, and self-management or home remedy. Female and/or elderly participants were more likely to seek NCD services from nearby primary-level facilities, including community clinics (CCs), family welfare centers (FWCs), and Union Health Centers (UHCs). Male participants were more likely to go to the Upazila Health Complex (first-level hospital) or upper-level healthcare facilities. One KII participant from Jhenaidah stated:

*Female and/or elderly people are more likely to come to nearby healthcare facilities. The CCs and FWCs are the nearest healthcare facilities located in the village or ward levels. They are comfortable visiting facilities that are located a short distance away.*

The level of knowledge, health service information, and perceived need for NCD services influenced care-seeking behavior and practice. Over two-thirds of the FGD participants lacked sufficient and accurate information about NCDs (e.g., types, symptoms, management). The majority of participants possessed limited information about NCD types, risks, and consequences of four major NCDs. A lack of information about NCD-related risks and consequences often limits people’s willingness to seek the necessary services available at PHC facilities. A female FGD participant from Jhenaidah stated:

*I had a feeling of extreme fatigue, shortness of breath, and dizziness. I had upper body discomfort and chest pain. I thought these were happening due to gastritis and took medicine to reduce it from a local drug outlet. But my pain and discomfort come and go. Once I visited a doctor at UHC and learned I had high blood pressure and high cholesterol. It had developed for a long time, but I did not recognize it.*

However, in some cases, the participants recognized the symptoms of diabetes (e.g., loss of appetite, frequent urination, thirstiness) and CVD (high blood pressure, fatigue) but did not realize their consequences nor the need to take preventive measures or seek treatment. A male participant from the FGD in Sylhet stated:

*I developed high blood pressure over the last few years, but it might not make me ill and affect my normal life. I do not pay much attention to it as it is normal.*

A similar observation was stated by a KII participant from Rajshahi:

*In many cases, NCD patients are asymptomatic. They don’t understand that they have high pressure. They may come to the health facilities for other diseases and are diagnosed with diabetes, high blood pressure, cholesterol, and heart diseases. The lack of realizing the symptoms and risk factors of diseases may lower the preference and willingness to receive healthcare services from the primary level facilities around them.*

This lack of adequate knowledge about symptoms and consequences often impeded them from seeking preventive service and care, which is the key strength of PHC facilities. Usually, the people sought curative care. A KII participant from Jhenaidah observed:

*Community people hardly realize the importance of preventive services. Prevention is the key to better managing NCDs. Early detection, awareness building for lifestyle modification, and timely referral are important parts of preventive services. But people usually visit the healthcare center to seek these services due to the misperception that healthcare services are curative services.*

Additionally, a lack of sufficient information about the available healthcare providers, facilities, and resources (e.g., medicine and equipment) resulted in a low preference and willingness to use NCD services in PHC facilities. A few participants perceived that the PHC system focused on the management of common illnesses and had limited or no services for NCDs such as respiratory illness, heart disease, cervical cancer, and diabetes. A male participant in the FGD in Sylhet quoted:

*Primary healthcare facilities are for managing common colds, fever, coughing, and headaches. They will give you a few iron tablets or gastric tablets. That’s it.*

Some participants perceived that PHC facilities were more suitable for managing diseases that mostly affected women (e.g., reproductive health, family planning, ante-natal, and post-natal care) and children, and people who needed services and care were more likely to attend PHC facilities. A participant stated:

*Most delivery patients and females, specifically married females, are more willing to go to primary healthcare facilities. Except for these, if NCD patients go there, they don’t give any importance as it is a female-focused healthcare service. (A male participant from the FGD in Cumilla)*

Misperception was a common factor that hindered the preference and willingness of individuals in seeking necessary services from PHC facilities. The majority of FGD participants thought that NCDs were diseases or conditions that affected the elderly (aged 60 years and above). Young people were not at risk and, therefore, were less willing to seek care. A participant stated:

*It’s believed young people aged below 60 are unlikely to develop NCDs like diabetes, heart diseases, and respiratory diseases. Thus, young people have little or no chance of seeking NCD services from nearby healthcare facilities. (A female participant from the FGD in Rajshahi)*

Similar views were shared by the KII participants:

*There is a lot of misconception regarding NCDs. Many people believe young people under the age of 50 have less chance of developing NCDs, which is not true in many cases. In many cases, young people have symptoms of diabetes, asthma, or heart disease, but they are reluctant to visit nearby primary healthcare facilities. Thus, the condition gets worse, and when they visit upper-level healthcare facilities they need modern and specialized care. (A KII participant from Jhenaidah)*

Self-management appeared to be common practice in managing NCDs across all methods and sites. A participant mentioned that, on many occasions, people are reluctant to use services from healthcare facilities and prefer self-management at home. Self-management was widely practiced using herbal plants, creepers, or anything similar that was available in the community. The use of herbal plants and creepers was more commonly used for managing DM and CRI. A KII participant from Sylhet shared:

*Self-management or home remedy for NCDs, including respiratory illness, diabetes, and heart disease, are long-standing practices in our country that limits the willingness to visit nearby healthcare facilities on many occasions.*

### Interpersonal or family factors

On several occasions, the preference for using healthcare services at PHC facilities was influenced by family tradition, practice, and experience. Elderly people often preferred home-based self-management using traditional medicine (e.g., herbal, self-medication). In a few cases, the elderly and influential family members thought that NCD-related conditions might be better managed using traditional methods. These beliefs and thoughts were passed through generations. A female FGD participant in Sylhet explained:

*I have diabetes. I eat plant leaves, kalojira (black cumin), and methi (dried fenugreek leaves) as my mother advised. I think these are good for managing diabetes, as senior family members had experienced this for a long time.*

Another female participant from the FGD in Cumilla shared the same information:

*Our parents and grandparents are used to following traditional methods for treating a wide range of illnesses. I think these methods are good and useful for our generation too.*

However, families’ financial capacity was mentioned as a common factor in choosing facilities or providers of NCD services. Those who had greater financial capacity (e.g., affluent families) preferred to visit upper-level healthcare facilities as the first contact. Individuals from relatively low and/or middle-income families were more likely to choose services from their community, which usually included PHC facilities. A female participant from the FGD in Sylhet stated:

*Wealthy family members are more likely to prefer secondary or tertiary-level healthcare facilities as their first contact because they like to attain high-quality care. They may have little or no trust in primary healthcare facilities*

Another female participant from FGD in Cumilla explained:

*Wealthy families prefer to get care from private hospitals or clinics. Healthcare services at private healthcare facilities involve a relatively higher cost, but they may consider the quality of care.*

To some extent, participants were influenced by their peers or close acquaintances (e.g., colleagues, neighbors) when preferring to receive NCD services. Referent opinions greatly influenced those who had newly developed NCDs. In many cases, the referents recommended the facility or service that they trusted the most, which considerably influenced the preference for NCD-related services. One of the female KII participants from Jhenaidah explained:

*When people get sick, they seek advice or information from their peers or close friends regarding healthcare facilities and options that influence their health-seeking.*

### Community and social factors

The widespread presence of alternative and complementary healthcare providers or methods appeared to be a crucial factor in the likelihood of using PHC facilities for managing NCDs. Participants from all sites and methods mentioned that people with NCDs or risk factors (e.g., hypertension, chest pain, cough/mucus) usually visited nearby drugs outlets to purchase medicine on their own or according to the salesperson’s advice. Drug sale outlets are densely situated at all sites. It was common practice for drugs salespersons to provide advice on a range of medicines for treating DM, CRI, and CVD. A male participant from the FGD in Cumilla informed us:

*Drugs outlets are the most available physical structure around us. Drugs salespersons are well-known. They are welcoming and knowledgeable about many diseases or complications. They are available at any time and charge no fees for advising about medicines. We prefer to visit them as a point of contact.*

This view was also shared by a female KII participant in Sylhet:

*Drugs salespersons are well-known and charge no fees for recommending medicines that may attract people from the community.*

The village doctor (or quack) was the most popular first contact point for seeking NCD services. Sometimes, quacks offered diagnostical tests (checking blood sugar or blood pressure) or prescribed medication. They usually came from the same communities and were well-known to community members. They played a greater role in taking NCD patients to upper-level healthcare facilities (mostly private facilities or professionals) or private consultants. Quacks had a good connection with hospitals, clinics, and private practitioners. On many occasions, NCD patients preferred to visit the quack and seek advice for further steps. The quack often prescribed medication and/or recommended private hospitals, clinics, or consultant physicians, if needed. A male participant from a FGD in Sylhet reflected:

*We go to the village doctors. If they say, “I can’t treat it, go to the city,” then we go there.*

One of the KII participants from Jhenaidah stated:

*Village doctors are the most common and influential health service providers in our context. They provide service through generations. People often trust, rely on, and accept their services to a great extent.*

A wide range of informal providers, including *hakem, kabiraj, ojha,* and *faith leaders* was mentioned as potential and popular NCD-related service providers or professionals. Over two-thirds of the participants stated that informal professionals/providers were easily accessible and provided effective care at a minimal cost. NCD patients from low- and middle-income families were more likely to choose informal providers. A few participants mentioned that NCD patients with complicated conditions (e.g., asthma or paralysis) who were involved in long-term family and home-based care and support preferred to seek care from informal care providers. A male FGD participant from Cumilla mentioned;

*Traditionally, informal providers have greater acceptance in our society. Many people believe that they can provide good care and manage complicated cases like cancer or other chronic conditions. People prefer to seek care from them instead of qualified doctors due to their beliefs.*

Another male participant from the FGD in Jhenaidah explained:

*Many families trust kabiraj, ojha, and faith healers to manage NCDs. They may offer care at minimal cost with confidence. They are usually overconfident about their service and assure the patients at the highest level, which may attract many of us.*

However, a few participants mentioned that the influence and acceptance of traditional providers have decreased in recent times as people are more aware of modern medicine. A female FGD participant from Jhenaidah stated:

*They were popular in the past. But, nowadays, people avoid them.*

### Organizational and health system factors

Numerous participants mentioned that their preference and willingness to receive NCD services from PHC facilities were influenced by the extent of the facilities or services available or offered. Insufficient supplies and facilities, including medicines, diagnostic capacity, and trained healthcare professionals were commonly mentioned as the influencing factors in choosing PHC facilities. Several participants thought that PHC facilities had a very limited scope for providing the necessary NCD services and care. Although the availability of PHC facilities was viewed positively, resources, procedures, and practices were identified as barriers to effective NCD management. The participants thought that insufficient resources and supplies resulted in the community avoiding primary-level facilities; a shortage of medicine was frequently mentioned. A male participant from a FGD in Sylhet claimed:

*If I go to the union level facilities at Union Sub-Center, they will give me only paracetamol and napa tablets. But I have to spend 50 BDT (1 BDT ≅ 87 USD) on the travel fare. I can buy a few packets of tablets with this travel fare.*

Similar views were shared regarding the diagnostic capacity of PHC facilities:

*I have diabetes and need to do some diagnostic tests, but the primary-level facilities do not have that capacity. I prefer to go to private facilities as they have modern instruments. (A male participant from a FGD in Jhenaidah)*

However, some KII participants mentioned a notable increase in medicine supplies at PHC facilities, including CCs and UHCs, but many patients who seek care from private facilities or professionals come from the government facilities to get medicine as they are free. A KII participant from Sylhet stated:

*Some patients prefer to seek care from private facilities but come to the government facilities to get medicine as it is free. I acknowledge that the supply of medicine is still insufficient, but they have increased significantly in the past few years. Over 20 types of medicine are provided in primary-level care facilities. It should be appreciated.*

A higher number of participants revealed that they appreciated the location of PHC facilities within a short distance (5–10 km) from the household that had good travel facilities. However, the situation in Sylhet was different because of some natural barriers (e.g., rivers, haors, and hills) that restricted people’s movements in seeking healthcare. In this case, NCD patients opted for alternative services such as home-based self-management or receiving care from informal providers who were easily accessible. A male participant of a FGD in Sylhet explained:

*It takes almost two hours to get to the hospital which involves higher travel costs. Sometimes, it is better to visit a traditional provider in the community to save time and money.*

The cost of treatment was considered when choosing services and facilities. Most of the participants stated that PHC facilities were relatively cheap and were favorable destinations as the first point of contact for NCD services. However, long wait times, mismanagement, and overcrowding were barriers to using the primary-level care facilities. Many participants mentioned that overcrowding and long wait times were very common in the Upazila Health Complex. A female FGD participant in Jhenaidah mentioned:

*The procedure is very slow in Upazila Health Complex. I have to take a ticket first, wait for a long time; it kills time. Say, I have taken the ticket at 10 a.m., and the doctor will see me at 1 p.m. after completing the round of indoor visits. Therefore, I have to sit until 1 p.m. I don’t have much time. Therefore, I went to private medical.*

The quality of services in PHC facilities is notably mentioned as the biggest limitation of the existing system. The majority of participants thought that the quality of services provided at primary-level facilities was of a lower standard, and because of this, many patients considered seeking care from secondary or upper-level facilities; therefore, private healthcare facilities became more preferable for seeking NCD services. A male participant in the FGD in Rajshahi mentioned:

*Actually, neither in Durgapur nor in Rajshahi will you get better treatment. Better treatment is available in a private clinic.*

Using common medicines, short consultation times with patients, little or no diagnostic capacity, and unempathetic attitudes of healthcare professionals were commonly mentioned factors that resulted in poor quality of care. Many participants viewed the quality and skills of healthcare professionals at PHC facilities as the cause of the poor quality of care. In many cases, this influenced patients to avoid primary-level facilities and seek care from an alternative provider (mostly private and upper-level facilities). However, the KII participants shared different views, as they thought people had misconceptions about the quality of care in that many people believed that quality care meant overprescribing (e.g., irrational use of antibiotics) or using ultra-modern technology. A KII participant in Rajshahi reflected:

*It is a trend to claim that government facilities do not provide quality care. People like to visit a good doctor, but who are the good doctors? People think good doctors are those who sit in private clinics with more patients. People have little or no trust in government facilities because they do not give irrational prescriptions. Suppose the patient needs paracetamol but expects azithromycin. Those who give are good doctors and provide good quality treatment. They have this mentality. The doctor has given me normal medicine; my problem will surely not be cured with it.*

The referral system was viewed as ineffective or absent, resulting in the overuse of some healthcare facilities and vice-versa. The participants stated that there was no functional referral system in the healthcare system in Bangladesh, and as a consequence, any patient could seek healthcare services from any facility or professional according to their needs, choice, and ability. In many cases, individuals irrationally choose higher-level facilities through personal choice or affordability. Patients with greater financial capacity often prefer private consultations and healthcare facilities for very minor issues (e.g., measuring blood pressure). A male participant in the FGD in Rajshahi mentioned:

*The treatment quality in the Upazila healthcare facilities is very bad. We go directly to Rajshahi (secondary and tertiary-level facilities).*

## Discussion

Using socio-ecological framework, this study explored the factors influencing preference and willingness to receive NCD services and care from PHC facilities in Bangladesh, and the results revealed that a wide array of factors at individual, interpersonal, societal, and organizational levels were interlinked and influenced each other in this respect. There has been an increasing interest in the use of different frameworks on different levels to investigate individuals' intentions and behavior in the utilization of health services. As such, the health belief model is widely used to explain and predict an individual’s health behavior but is viewed as having a limited capacity to comprehensively explain complex interactions between behavior and structural and/or environmental influences [33]. Interpersonal framework (e.g., social cognitive theory, social network theory) explains the social influence on behavior with the difficulty in operationalizing interventions [34]. Andersen’s behavioral model has been extensively applied to investigate the use of health services [35]. Over the past decades, this model has undergone continuous development that placed various factors from individual to environmental levels and was noticeably modified in the context of the specific target group, diseases, and settings. Currently, multiple versions of models are being used in health service research [36]. It suggests that peoples’ access to healthcare services is determined by an array of individual, contextual characteristics, and health needs. However, this model has been paying limited attention to social networks and interactions, and culture [37].

To provide a more comprehensive multilevel framework, the socio-ecological framework was used as an analytical and conceptual tool to explore and interpret factors that influence the preference and willingness to use NCD services from PHCs. This framework has been considered the best suited for the current study as it helped to conceptualize health behavior as a result of a dynamic interplay of multiple factors between individual, group/community, institutional, societal, and organizational levels [30]. This framework was useful in shaping the research question and data analysis regarding the preference and willingness to use NCD services from PHCs.

An individual’s age, gender, healthcare-related information and perception, misconception, and scope of self-management were common factors that influenced the likelihood of using the services available at PHC facilities. The primary healthcare system includes a wide range of healthcare facilities within a vast infrastructural network [22, 38] that has increased the scope and coverage of NCD services over the years [39]. In particular, the number of healthcare facilities has increased over the last decade, notably through setting up CCs at the ward or village level [40]. Yet, CC services remain under-utilized due to misperceptions, a lack of awareness, and a lack of proper healthcare information [41, 42]. Health-related information, such as symptoms of illness, perceived severity, and possible consequences, are important determinants in seeking care [43, 44]. Our findings suggest that a community’s perceived poor level of knowledge of symptoms and risk factors, neglected preventive and promotive care, and possible complications of major NCDs result in a delay in or avoidance of timely care-seeking from PHC facilities. Avoiding or delaying care often results in the condition or disease becoming more complicated, requiring specialized and modern treatment facilities that are unavailable in the primary healthcare system [5]. The roles and scope of the primary healthcare system for NCDs were viewed narrowly by community members. There was a view that PHC facilities provided treatment to cure diseases. This view would have limited the chance to receive promotional and preventive healthcare support, which was emphasized in the Alma–Ata Declaration and major policy documents [45, 46].

The influence of family members in seeking care has been a long-held tradition in Bangladesh society. The decision of choosing care providers or facilities is often influenced by the male and/or elderly person in the family. The elderly male, who is usually the head of the household, was the most influential in preferencing and choosing services based on the perceived severity of disease, affordability (financial capacity), and healthcare information. In severe conditions, the family considered the relatives’ and close friends’ views and opinions. Wealthy families did not choose primary-level care facilities but preferred secondary or tertiary-level facilities, even for treating common illnesses or monitoring routine glucose levels. Opinions and healthcare information were important in choosing care providers because the severity of the condition or disease and an individual’s knowledge and perception appeared to be influencing factors.

In terms of community and societal factors, frontline healthcare providers, such as health assistants and family welfare assistants, influenced the use of services at PHC facilities. Patients sometimes sought care from the facilities around them (e.g., community clinics) at the onset of symptoms or risk factors (e.g., breathing difficulties, raised blood pressure) or in the initial stages of severe disease or conditions (e.g., diabetes). A higher proportion of healthcare providers were from informal sectors, including village doctors, spiritual healers (*hujur* or *trantrik*), traditional healers (*kabiraj* or *ojha*), and drugs salespeople who played important roles in treating illnesses in Bangladeshi society [47–49]. Our findings are concordant with previous studies that show that village doctors and/or drugs salespeople are highly accessible and popular for treating illness. Their widespread presence, proximity to the community, and low-cost treatment (they usually do not charge consultation fees) helped them gain great acceptance as they appeared to be pro-poor service providers in the Bangladesh context [25, 47]. These doctors and salespeople usually came from the same community and traditionally provided services and care for different generations; therefore, community norms and traditions may have passively influenced their roles and helped them achieve a greater level of acceptance.

Health system factors greatly influence the utilization of healthcare services. The greater availability of equipment and resources, the attitude of service providers, and the geographical proximity of healthcare facilities greatly influenced the preference and willingness to seek healthcare [50–52]. Over the past few years, the availability of PHC facilities has noticeably increased in Bangladesh via the set-up of CCs [53, 54]. However, our findings indicated that people living with NCDs expressed a low level of preference and willingness for using NCD services. The provision of healthcare services in CCs was focused more on managing family planning services, reproduction and child care, and basic health services, which might have influenced their preference and willingness [55]. The shortage of supplies and resources, including medicines and diagnostic facilities in CCs, UHCs, and Upazila Health Complex resulted in a lower preference for using NCD services, which is similarly reported in primary healthcare settings in several countries [56–59]. The cost, physical distance, and wait time also influenced the utilization of PHC facilities. Although studies conducted in resource-poor settings indicated that long-distance and cost were significant factors for choosing healthcare services, our findings indicated that the presence of healthcare facilities with affordable costs were positive factors in the primary healthcare system. However, the perceived quality of healthcare in PHC facilities was notably lower, which resulted in a lower preference and willingness to seek care from these facilities. Quality of healthcare is an important predictor of service utilization across the globe [60–62]. The lack of a proper referral system noticeably influenced rational and need-based utilization of healthcare services. An ineffective referral system resulted in excessive use of secondary or tertiary-level care facilities. There is no structural or functional referral system in existing healthcare that allows patients to directly seek care from higher-level healthcare facilities to manage even mild and general ailments [63]. The lack of a proper referral system allowed patients to bypass PHC facilities in the study [64].

### Strengths and limitations

The major strength of this study is the inclusion of participants from a wide range of professions, ages, and gender, which enabled the optimization of variations of participants. Another strength is the use of multiple data collection tools that allowed us to triangulate methods and participants to increase the trustworthiness of the findings. Furthermore, applying a SEM offered an analytical basis for the key individual, interpersonal, social, and organizational elements that influenced the preference and willingness to receive services from primary-level care facilities in preventing and managing NCDs. A possible limitation is that some participants might have been uncomfortable sharing their opinions and thoughts in the group discussions due to dominant participants. However, the experienced and trained facilitators tried to minimize this limitation by maintaining good group dynamics during the discussions. The findings of this study are based on data from rural settings; therefore, the results may not be easily generalizable across populations and places. Nevertheless, information collected from a diverse group of participants was sufficient to provide an in-depth understanding of factors influencing the preference and willingness to receive services from primary-level care facilities in preventing and managing NCDs.

## Conclusion

This study revealed that a broad range of factors at different levels (individual, interpersonal, social, and organizational) influenced the preference and willingness of community members to use services from primary-level healthcare settings in preventing and managing NCDs. The factors are interconnected and influence each other. Despite the remarkable increase and expansion of primary healthcare services, the preference and willingness to use the services remain low. Individual factors, such as age and gender, misconception about NCDs and related services, interpersonal factors like family practices and financial capacity, social factors, including traditional practices and beliefs, the widespread presence of informal providers, and organizational factors, such as insufficient supplies and commodities, poor quality of services, and a weak referral system hinder the full potential of the primary healthcare system in preventing and managing the increasing burden of NCDs. To promote the preference and willingness to use services in PHC facilities, different strategies are needed to address the influential factors. However, special attention needs to be paid to the individual (a lack of awareness about NCDs and related services) and organizational levels (quality of care and the presence of unregulated alternative healthcare providers) as they are the most vital factors that influence the use of primary healthcare services.

## Data Availability

The data used and analyzed during this research are not publicly available due to ethical restrictions, and data confidentiality. Data are available upon reasonable request from researchers who meet the criteria for access to confidential data. Interested parties may contact the first author (md.kabir@monash.edu) for further inquiries in this regard.

## Abbreviations

BMRC: Bangladesh Medical Research Council
CCs: Community clinics
CRI: Chronic respiratory disease
CVDs: Cardiovascular diseases
COREQ: Consolidated criteria for reporting qualitative studies
DM: Diabetes mellitus
FGDs: Focused group discussions
FWCs: Family welfare centers
KIIs: Key informant interviews
NCDs: Non-communicable diseases
PHC: Primary healthcare
SEM: Social-ecological model
UHC: Upazila Health Complex
WHO–PEN: World Health Organization’s package of essential noncommunicable disease interventions
